# Associations of lipid accumulation product, visceral adiposity index, and triglyceride-glucose index with subclinical organ damage in healthy Chinese adults

**DOI:** 10.3389/fendo.2023.1164592

**Published:** 2023-09-19

**Authors:** Ming-Fei Du, Xi Zhang, Gui-Lin Hu, Jian-Jun Mu, Chao Chu, Yue-Yuan Liao, Chen Chen, Dan Wang, Qiong Ma, Yu Yan, Hao Jia, Ke-Ke Wang, Yue Sun, Ze-Jiaxin Niu, Zi-Yue Man, Lan Wang, Xiao-Yu Zhang, Wen-Jing Luo, Wei-Hua Gao, Hao Li, Guan-Ji Wu, Ke Gao, Jie Zhang, Yang Wang

**Affiliations:** ^1^ Department of Cardiovascular Medicine, First Affiliated Hospital of Xi’an Jiaotong University, Xi’an, China; ^2^ Key Laboratory of Molecular Cardiology of Shaanxi Province, First Affiliated Hospital of Xi’an Jiaotong University, Xi’an, China; ^3^ Department of Cardiology, Xi’an International Medical Center Hospital, Xi’an, China; ^4^ Department of Cardiology, Northwest Women’s and Children’s Hospital of Xi’an Jiaotong University Health Science Center, Xi’an, China; ^5^ Department of Cardiology, Xi’an No.1 Hospital, Xi’an, China; ^6^ Department of Critical Care Medicine, First Affiliated Hospital of Xi’an Jiaotong University, Xi’an, China; ^7^ Department of Cardiology, Xi’an Central Hospital of Xi’an Jiaotong University, Xi’an, China; ^8^ Department of Cardiology, Xi’an People’s Hospital, Xi’an, China

**Keywords:** lipid accumulation product, visceral adiposity index, triglyceride-glucose index, albuminuria, arterial stiffness, left ventricular hypertrophy, subclinical organ damage

## Abstract

**Background and aims:**

Obesity is an independent risk factor for cardiovascular disease development. Here, we aimed to examine and compare the predictive values of three novel obesity indices, lipid accumulation product (LAP), visceral adiposity index (VAI), and triglyceride-glucose (TyG) index, for cardiovascular subclinical organ damage.

**Methods:**

A total of 1,773 healthy individuals from the Hanzhong Adolescent Hypertension Study cohort were enrolled. Anthropometric, biochemical, urinary albumin-to-creatinine ratio (uACR), brachial-ankle pulse wave velocity (baPWV), and Cornell voltage-duration product data were collected. Furthermore, the potential risk factors for subclinical organ damage were investigated, with particular emphasis on examining the predictive value of the LAP, VAI, and TyG index for detecting subclinical organ damage.

**Results:**

LAP, VAI, and TyG index exhibited a significant positive association with baPWV and uACR. However, only LAP and VAI were found to have a positive correlation with Cornell product. While the three indices did not show an association with electrocardiographic left ventricular hypertrophy, higher values of LAP and TyG index were significantly associated with an increased risk of arterial stiffness and albuminuria. Furthermore, after dividing the population into quartiles, the fourth quartiles of LAP and TyG index showed a significant association with arterial stiffness and albuminuria when compared with the first quartiles, in both unadjusted and fully adjusted models. Additionally, the concordance index (C-index) values for LAP, VAI, and TyG index were reasonably high for arterial stiffness (0.856, 0.856, and 0.857, respectively) and albuminuria (0.739, 0.737, and 0.746, respectively). Lastly, the analyses of continuous net reclassification improvement (NRI) and integrated discrimination improvement (IDI) demonstrated that the TyG index exhibited significantly higher predictive values for arterial stiffness and albuminuria compared with LAP and VAI.

**Conclusion:**

LAP, VAI, and, especially, TyG index demonstrated utility in screening cardiovascular subclinical organ damage among Chinese adults in this community-based sample. These indices have the potential to function as markers for early detection of cardiovascular disease in otherwise healthy individuals.

## 1 Introduction

Cardiovascular disease is a growing public health burden on the global population because of its increasing morbidity and mortality (1). Furthermore, metabolic abnormalities, which are prevalent in obesity, serve as independent risk factors for cardiovascular disease. Studies have reported that obesity at any time in life is independently associated with increased albuminuria risk, irrespective of obesity-related comorbidities, including hypertension, dyslipidemia, and impaired glucose tolerance (2, 3). Moreover, obesity is an established risk factor for developing arterial stiffness and left ventricular hypertrophy (LVH), which in turn have been suggested as early detectable measures of cardiovascular disease and are associated with related long-term adverse outcomes, such as coronary heart disease, stroke, and sudden cardiac death (4, 5).

The obesity-related adverse outcomes are not simply concerned with the extent but also the distribution (6). Previous evidence has demonstrated that visceral adipose tissue rather than subcutaneous adipose tissue may play an important role in systemic injuries, such as those in blood vessels, heart, and kidneys, making it an independent marker of cardiovascular and metabolic morbidity and mortality (7, 8). Although body mass index (BMI) is a powerful predictor of obesity, visceral fat accumulation may vary among people with the same BMI. Waist circumference (WC) is the easiest and the most common index used for evaluating central obesity in clinical practice, replacing BMI in the clinical diagnosis of metabolic syndrome (9). However, WC cannot efficiently distinguish visceral fat from subcutaneous fat, and subcutaneous fat is a much weaker indicator of cardiovascular risk (10). Additionally, computed tomography and magnetic resonance imaging are the gold methods for evaluating and quantifying fat distribution and have developed rapidly and tended to maturely (11, 12). However, they are not routinely applied in clinical settings and community screening due to their high cost, radiation exposure, and operation complexity.

Three reliable markers of visceral adiposity were proposed based on anthropometric and metabolic parameters. First, lipid accumulation product (LAP) is an index that combines WC and fasting triglyceride (TG) values (13). LAP has shown potential for predicting the risk of hypertension, renal function decline, impaired fasting glucose, and even diabetes and cardiovascular disease, while outperforming BMI (14–18). Second, visceral adiposity index (VAI) is a measurement estimated using TG and high-density lipoprotein cholesterol (HDL-C) levels as well as BMI and WC values (19). Finally, the triglyceride-glucose (TyG) index is calculated by incorporating TG and fasting blood glucose (FBG) levels. The TyG index has exhibited associations with insulin resistance, arterial stiffness, and cardiovascular disease (20, 21). Previous studies have primarily focused on evaluating the value of single surrogate markers of visceral adiposity, such as LAP, VAI, and TyG index, to identify the risk for single cardiovascular subclinical organ damage and even cardiovascular disease. However, the performance of all three markers in predicting subclinical organ damage (SOD) remains unclear.

Therefore, in this study, we conducted a cross-sectional analysis based on our previously established Hanzhong cohort to investigate and compare the associations of LAP, VAI, and TyG index with SOD, including albuminuria, arterial stiffness, and LVH, in Chinese adults. We further aimed to examine the predictive capacity of the three indices for SOD in the general population.

## 2 Materials and methods

### 2.1 Study cohort

The study population was derived from the Hanzhong Adolescent Hypertension Study cohort, established in 1987. A total of 4,623 children and adolescents were enrolled from 26 rural areas in three towns in Hanzhong City, Shaanxi Province, China. The ongoing prospective, population-based cohort study included regular screenings to monitor the development of cardiovascular risk factors. The study protocol details have been published elsewhere (22, 23).

Here, we explored the association of LAP, VAI, and TyG index with the risk of SOD using the cross-sectional analysis of the latest follow-up data in 2017. The flowchart of the participant selection process is outlined in **Figure S1**. A total of 2,780 participants were followed up in 2017, yielding a response rate of 60.9%. Participants with missing data on serum or urinary biochemistry (n = 147 and 390, respectively), anthropometric data (n = 29), and brachial-ankle pulse wave velocity (baPWV) and LVH data (n = 474) or a self-identified history of coronary heart disease, severe arrhythmia, renal failure, or stroke (n = 7) were excluded, leaving 1,733 participants available for our primary analysis.

### 2.2 Clinical and anthropometric measurements

Participants completed on-site questionnaires that collected general information on their smoking habits, alcohol consumption, physical activity, medical conditions, and medication history. Physical examinations included body height, weight, and WC. BMI was calculated as weight (kg) divided by the square of height (m^2^). Blood pressure (BP) was measured in the sitting position using a standard mercury sphygmomanometer. The average of three BP readings was recorded. The baPWV was assessed using an automatic arteriosclerosis diagnostic device (BP-203RPEII; Nihon Colin, Tokyo, Japan). Briefly, four cuffs were wrapped around the upper arms and ankles of the supine participants and connected to plethysmographic and oscillometric pressure sensors. The highest baPWV value obtained was included in the analysis. Further details of the baPWV measurement process have been described previously (24). A routine 12-lead electrocardiogram (ECG) examination was conducted, as reported earlier (23), with a paper speed of 25 mm/s and a gain of 10 mm/mV.

### 2.3 Biochemical assays

Blood samples were acquired in the morning after at least 8 h of fasting. The blood samples were immediately centrifuged to separate the serum, which was stored at −80°C. Spot urine samples were obtained from the first urine in the morning and frozen at −40°C. Biochemical parameters, including FBG, TG, total cholesterol, low-density lipoprotein cholesterol (LDL-C), HDL-C, creatinine, uric acid, and albumin, were measured as reported previously (24). The estimated glomerular filtration rate (eGFR) was calculated as follows: eGFR (mL/min per 1.73 m^2^) = 175 × serum creatinine (mg/dL)^−1.234^ × age (years)^−0.179^ (× 0.79 for women) (25). Furthermore, the three visceral adiposity parameters were calculated using the following formulas (26, 27): LAP = (WC [cm] − 65 for men [58 for women]) × TG (mmol/L), TyG index = ln (TG [mg/dL] × FBG [mg/dL]/2), VAI for men = (WC [cm]/[39.68 + 1.88 × BMI {kg/m^2^}]) × (TG [mmol/L]/1.03) × (1.31/HDL-C [mmol/L]), VAI for women = (WC [cm]/[36.58 + 1.89 × BMI {kg/m^2^}]) × (TG [mmol/L]/0.81) × (1.52/HDL-C [mmol/L]).

### 2.4 Definitions

Hypertension was defined as systolic blood pressure (SBP) of ≥140 mmHg, diastolic blood pressure (DBP) of ≥90 mmHg, or the present use of antihypertensive drugs (28). Diabetes was described as FBG of ≥7.0 mmol/L, the current use of antidiabetic medication, or a previous history of diabetes (29). Hyperlipidemia was considered as the occurrence of hypertriglyceridemia (TG ≥2.26 mmol/L), hypercholesterolemia (total cholesterol ≥6.22 mmol/L), high LDL-C level (≥4.14 mmol/L), or low HDL-C level (≤1.04 mmol/L) (30). Participants were classified as having hyperuricemia if their serum uric acid levels were >420 μmol/L (in both men and women) (31). Albuminuria was defined as a urinary albumin-to-creatinine ratio (uACR) of ≥30 mg/g (32). Furthermore, increased baPWV has been reported to be a good indicator of arterial stiffness development, with a baPWV value of ≥1,400 cm/s considered a high-risk marker (33). ECG is a screening method for LVH, wherein the Cornell voltage-duration product (“Cornell product”) is one of the common parameters employed (28). The Cornell product (mV·ms) is calculated as follows: RaVL + SV3 (with 0.8 mV added in women) × QRS duration, with a Cornell product of ≥244 mV·ms indicating ECG-LVH (34).

### 2.5 Statistical analyses

According to their distribution, continuous variates were presented as means ± standard deviations (SDs) or medians (25^th^ and 75^th^ percentiles). Statistically significant differences among groups were calculated using one-way analysis of variance, Mann–Whitney U test, Kruskal–Wallis H test, and *post-hoc* test as appropriate. Linear trends were tested by the Jonckheere–Terpstra test. The Spearman correlation coefficient was employed to analyze the association between two variates. Categorical variates were expressed as frequencies (n) or percentages (%). The χ^2^ test was applied to assess the differences, whereas the linear-by-linear association test was performed to estimate the linear trends. Multivariable linear and logistic regression analyses were conducted to determine the odds ratios (ORs) and 95% confidence intervals (95% CIs) for the predictive effect of the risk factors on SOD, with adjustments for age, sex, SBP, serum creatinine, physical activity, smoking habit, and alcohol consumption. Three logistic regression models were constructed to evaluate the association of LAP, VAI, and TyG index with SOD risk. After the initial unadjusted analysis, age and sex were used as adjustment covariates in model 1. Next, SBP, serum creatinine, physical activity, smoking habit, and alcohol drinking were included as covariates in model 2. Additionally, all covariates were examined for multicollinearity before multivariate analysis. We further conducted subgroup analysis to assess the robustness of the associations of LAP, VAI, and TyG index with the risk for SOD. The predictive values of the three indices for SOD were estimated by calculating their concordance index (C-index) based on the fully adjusted model. According to this metric, C-index values over 0.7 indicate a good model (35). To further evaluate the discriminative ability of the indices, we performed pairwise comparisons of the fully adjusted models with LAP, VAI, or TyG index, along with continuous net reclassification improvement (NRI) and integrated discrimination improvement (IDI) analyses. Statistical analyses were conducted using R software 4.2.2 (R Foundation for Statistical Computing, Vienna, Austria) and SPSS 25.0 (SPSS Inc., IL, USA). Statistical significance was set at a two-tailed *P* value of <0.05.

## 3 Results

### 3.1 Association of LAP, VAI, and TyG index with arterial stiffness risk

The characteristics of the participants (n = 1,733) grouped according to the presence or absence of arterial stiffness are shown in **Table 1**. Participants with arterial stiffness (n = 456) were more likely to be male and displayed higher LAP, VAI, TyG index, uACR, and Cornell product values than those without arterial stiffness (*P* < 0.05). Overall, participants with arterial stiffness had a more adverse metabolic and cardiovascular risk profile (**Table 1**).

**Table 1:** Characteristics of participants categorized by arterial stiffness status in 2017 (n = 1,733).

Characteristics	All (n = 1,733)	Non-AS (n = 1,277)	AS (n = 456)	*P* value
Gender (male, %)	980 (56.55%)	643 (50.35%)	337 (73.90%)	**<0.001**
Age (years)	43 (40-45)	43 (40-45)	44 (41-45)	**<0.001**
Diabetes mellitus (%)	395 (22.79%)	260 (20.36%)	135 (29.61%)	**<0.001**
Hypertension (%)	312 (18.00%)	140 (10.96%)	172 (37.72%)	**<0.001**
Hyperuricemia (%)	89 (5.14%)	49 (3.84%)	40 (8.77%)	**<0.001**
Hyperlipidemia (%)	694 (40.05%)	459 (35.94%)	235 (51.54%)	**<0.001**
Alcohol consumption (%)	509 (29.37%)	332 (26.00%)	177 (38.82%)	**<0.001**
Current smoking (%)	757 (43.68%)	495 (38.76%)	262 (57.46%)	**<0.001**
Obesity indices				
BMI (kg/m^2^)	23.78 (21.85-26.02)	23.38 (21.55-25.43)	24.98 (22.99-26.88)	**<0.001**
WC (cm)	84.20 (78.00-91.40)	82.90 (76.95-89.70)	88.36 ± 9.62	**<0.001**
LAP	29.77 (17.22-50.69)	26.38 (15.63-45.29)	41.35 (23.92-65.66)	**<0.001**
VAI	1.80 (1.17-2.76)	1.68 (1.10-2.57)	2.09 (1.40-3.17)	**<0.001**
TyG index	8.49 (8.14-8.90)	8.42 (8.09-8.80)	8.71 (8.34-9.10)	**<0.001**
Measurement indicators				
Heart rate (beats/min)	73 (66-80)	72 (69-79)	75 (69-83)	**<0.001**
SBP (mmHg)	121 (112-131)	117 (110-125)	134 (126-148)	**<0.001**
DBP (mmHg)	76 (69-84)	73 (67-79)	86 (80-94)	**<0.001**
FBG (mmol/L)	4.57 (4.28-4.90)	4.54 (4.26-4.85)	4.65 (4.33-5.06)	**<0.001**
ALT (U/L)	19 (14-27)	18 (13-26)	22 (15-33)	**<0.001**
AST (U/L)	16 (13-20)	16 (13-20)	17 (14-22)	**<0.001**
Total cholesterol (mmol/L)	4.50 (4.04-5.01)	4.47 (4.01-4.96)	4.64 (4.17-5.18)	**<0.001**
Triglycerides (mmol/L)	1.34 (0.95-1.94)	1.24 (0.91-1.80)	1.63 (1.15-2.30)	**<0.001**
LDL-C (mmol/L)	2.46 (2.11-2.84)	2.46 (2.10-2.84)	2.64 ± 0.67	**<0.001**
HDL-C (mmol/L)	1.15 (0.99-1.33)	1.17 (1.01-1.35)	1.09 (0.96-1.26)	**<0.001**
Serum UA (μmol/L)	279.90 (225.00-336.10)	268.60 (218.85-322.95)	314.19 ± 82.01	**<0.001**
Serum creatinine (μmol/L)	75.60 (66.45-85.90)	73.90 (65.30-84.75)	80.00 (71.13-87.65)	**<0.001**
eGFR (mL/min/1.73m^2^)	97.76 (87.60-110.77)	98.76 (88.19-111.75)	95.11 (85.48-108.09)	**0.006**
uACR (mg/g)	8.45 (5.52-14.72)	7.73 (5.09-12.95)	11.36 (6.85-24.86)	**<0.001**
baPWV (cm/s)	1,263.00 (1,126.00-1415.50)	1,190.00 (1,086.50-1,289.00)	1,543.00 (1,466.00-1,656.50)	**<0.001**
Cornell product (mV·ms)	138.60 (106.62-174.88)	137.50 (106.34-170.74)	149.00 ± 59.12	**0.014**

Non-normally distributed variables are expressed as the median (interquartile range). All other values are expressed as mean ± SD or n (%). AS, arterial stiffness; BMI, body mass index; WC, waist circumference; LAP, lipid accumulation product; VAI, visceral adiposity index; TyG index, triglyceride-glucose index; SBP, systolic blood pressure; DBP, diastolic blood pressure; FBG, fasting blood glucose; ALT, alanine aminotransferase; AST, aspartate aminotransferase; LDL-C, low-density lipoprotein cholesterol; HDL-C, high-density lipoprotein cholesterol; UA, urine acid; eGFR, estimated glomerular filtration rate; uACR, urine albumin-to creatinine ratio.

Statistically values are presented in bold.

**Figure 1:**
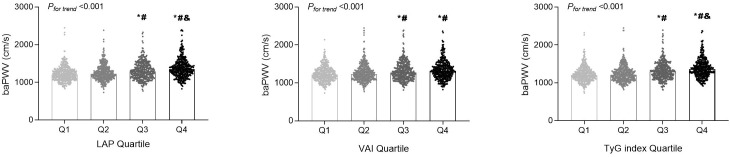
The distributions of the brachial-ankle pulse wave velocity (baPWV) values among the quartile (Q) groups according to the lipid accumulation product (LAP), visceral adiposity index (VAI), and triglyceride-glucose (TyG) index. ^*^P < 0.05 compared with the Q1 group; *^#^P* < 0.05 compared with the Q2 group; ^&^ *P* < 0.05 compared with the Q3 group.

In terms of baPWV, LAP, VAI, and TyG index values were positively correlated with baPWV values (*P* < 0.001 for all). We further assessed these associations by categorizing the distribution of the three parameters of visceral adiposity into quartiles (**Figure 1**). The values of baPWV in the third and fourth quartiles of LAP, VAI, and TyG index were significantly higher compared with the first and second quartiles. After adjusting for traditional cardiovascular risk factors and potential confounders, LAP (OR [95% CI] = 1.004 [1.001–1.007], *P* = 0.026) and TyG index (OR [95% CI] = 1.461 [1.165–1.831], *P* = 0.001) were independently associated with a higher risk for arterial stiffness (**Table 2**). Furthermore, the prevalence of arterial stiffness was significantly positive with the quartile of LAP, VAI, and TyG index (*P* < 0.001). As presented in **Table 3**, the third and fourth quartiles of LAP, VAI, and TyG index demonstrated notable associations with the presence of arterial stiffness, both before and after adjusting for age and sex, when compared with the first quartiles (model 1). After further adjustment (model 2), the fourth quartiles of LAP, VAI, and TyG index retained their high predictive values for the presence of arterial stiffness compared with the corresponding first quartiles (OR [95% CI] = 1.538 [1.032–2.291], *P* = 0.034; OR [95% CI] = 1.639 [1.111–2.417], *P* = 0.013; and OR [95% CI] = 1.778 [1.192–2.653], *P* = 0.005, respectively).

**Table 2:** Associations between various characteristics and subclinical organ damage in 2017 (n=1,733).

	Arterial stiffness		Albuminuria		ECG-LVH	
	OR (95% CI)	*P*	OR (95% CI)	*P*	OR (95% CI)	*P*
Gender (male, %)	1.888 (1.241-2.872)	**0.003**	0.401 (0.228-0.706)	**0.002**	0.292 (0.160-0.531)	**<0.001**
Age (years)	1.065 (1.019-1.113)	**0.005**	0.926 (0.878-0.977)	**0.005**	0.958 (0.908-1.011)	0.120
Current smoking (%)	0.942 (0.655-1.354)	0.746	1.673 (0.970-2.866)	0.064	1.179 (0.647-1.721)	0.591
Alcohol consumption (%)	1.069 (0.783-1.460)	0.673	0.604 (0.389-0.939)	**0.025**	1.061 (0.654-1.721)	0.811
BMI (kg/m^2^)	0.976 (0.933-1.022)	0.299	1.097 (1.043-1.155)	**<0.001**	1.041 (0.986-1.099)	0.145
WC (cm)	1.000 (0.985-1.015)	0.988	1.033 (1.014-1.052)	**0.001**	1.015 (0.996-1.034)	0.129
SBP (mmHg)	1.106 (1.094-1.119)	**<0.001**	1.051 (1.042-1.061)	**<0.001**	1.019 (1.009-1.028)	**<0.001**
Heart rate (bpm/s)	1.033 (1.019-1.046)	**<0.001**	1.025 (1.010-1.041)	**0.001**	0.984 (0.967-1.001)	0.059
ALT (U/L)	1.008 (0.999-1.018)	0.083	1.014 (1.003-1.025)	**0.011**	1.010 (0.998-1.022)	0.108
Serum UA (μmol/L)	0.449 (0.068-2.951)	**0.040**	1.001 (0.998-1.003)	0.588	1.002 (1.000-1.005)	0.088
Serum creatinine (μmol/L)	1.005 (0.994-1.016)	0.355	1.002 (0.989-1.014)	0.787	0.986 (0.973-1.000)	0.055
FBG (mmol/L)	1.094 (0.997-1.200)	0.058	1.415 (1.275-1.571)	**<0.001**	1.041 (0.923-1.175)	0.510
Triglycerides (mmol/L)	1.136 (1.029-1.254)	**0.011**	1.160 (1.041-1.292)	**0.007**	1.006 (0.864-1.172)	0.935
Total cholesterol (mmol/L)	1.188 (1.004-1.406)	**0.045**	1.089 (0.890-1.333)	0.407	0.990 (0.804-1.220)	0.928
LDL-C (mmol/L)	1.182 (0.957-1.460)	0.121	0.964 (0.741-1.255)	0.786	1.029 (0.786-1.345)	0.837
HDL-C (mmol/L)	0.902 (0.524-1.550)	0.708	0.651(0.328-1.292)	0.219	0.984 (0.510-1.901)	0.963
LAP	1.004 (1.001-1.007)	**0.026**	1.006 (1.003-1.010)	**<0.001**	1.002 (0.998-1.006)	0.337
VAI	1.047 (0.998-1.099)	0.062	1.068 (1.015-1.124)	**0.012**	1.010 (0.943-1.082)	0.768
TyG index	1.461 (1.165-1.831)	**0.001**	1.861(1.427-2.426)	**<0.001**	1.011 (0.757-1.350)	0.940

Models were adjusted for age, sex, smoking, alcohol consumption, physical activity, SBP, and serum creatinine. OR, odds ratio; 95% CI, 95% confidence intervals; ECG-LVH, electrocardiogram-left ventricular hypertrophy; BMI, body mass index; WC, waist circumference; SBP, systolic blood pressure; ALT, alanine aminotransferase; UA, urine acid; FBG, fasting blood glucose; LDL-C, low-density lipoprotein cholesterol; HDL-C, high-density lipoprotein cholesterol; LAP, lipid accumulation product; VAI, visceral adiposity index; TyG index, triglyceride-glucose index.

Statistically values are presented in bold.

**Table 3:** Association between various characteristics and arterial stiffness and albuminuria by multiple logistic regression analysis.

	Arterial stiffness				Albuminuria			
	n (%)	Odds ratios (95% confidence interval)			n (%)	Odds ratios (95% confidence interval)		
		Unadjusted	Model 1	Model 2		Unadjusted	Model 1	Model 2
LAP								
Quartile 1	66 (14.5%)	1 (reference)	1 (reference)	1 (reference)	28 (15.2%)	1 (reference)	1 (reference)	1 (reference)
Quartile 2	89 (19.5%)	**1.439 (1.013-2.043)^#^**	**1.536 (1.072-2.200)^#^**	1.163 (0.768-1.761)	39 (21.2%)	1.432 (0.864-2.372)	1.392 (0.839-2.311)	1.206 (0.712-2.044)
Quartile 3	128 (28.1%)	2.326 (1.666-3.247)^*^	2.235 (1.589-3.143)^*^	1.284 (0.859-1.920)	44 (23.9%)	1.632 (0.996-2.674)	**1.713 (1.043-2.813)^#^**	1.210 (0.715-2.049)
Quartile 4	173 (37.9%)	3.700 (2.673-5.121)^*^	3.424 (2.455-4.776)^*^	**1.538 (1.032-2.291)^#^**	73 (39.7%)	2.933 (1.855-4.638)^*^	3.204 (2.015-5.096)^*^	**1.889 (1.143-3.122)^#^**
*P* for trend	**<0.001**	**<0.001**	**<0.001**	**0.015**	**<0.001**	**<0.001**	**<0.001**	**0.017**
VAI								
Quartile 1	77 (16.9%)	1 (reference)	1 (reference)	1 (reference)	31 (16.8%)	1 (reference)	1 (reference)	1 (reference)
Quartile 2	99 (21.7%)	1.370 (0.982-1.912)	**1.483 (1.052-2.089)^#^**	1.330 (0.889-1.991)	39 (21.2%)	1.284 (0.785-2.099)	1.255 (0.766-2.054)	1.018 (0.607-1.706)
Quartile 3	129 (28.3%)	1.955 (1.419-2.696)^*^	2.079 (1.494-2.892)^*^	1.469 (0.990-2.181)	48 (26.1%)	**1.613 (1.005-2.587)^#^**	**1.607 (1.001-2.581)^#^**	1.179 (0.715-1.943)
Quartile 4	151 (33.1%)	2.476 (1.805-3.395)^*^	2.604 (1.881-3.604)^*^	**1.639 (1.111-2.417)^#^**	66 (35.9%)	2.332 (1.488-3.656)^*^	**2.352 (1.499-3.691)^#^**	1.555 (0.966-2.505)
*P* for trend	**<0.001**	**<0.001**	**<0.001**	0.079	**<0.001**	**<0.001**	**<0.001**	0.093
TyG index								
Quartile 1	70 (15.4%)	1 (reference)	1 (reference)	1 (reference)	34 (18.5%)	1 (reference)	1 (reference)	1 (reference)
Quartile 2	79 (17.3%)	1.157 (0.813-1.648)	1.084 (0.756-1.555)	1.011 (0.659-1.550)	35 (19.0%)	1.032 (0.631-1.688)	1.074 (0.655-1.759)	1.023 (0.610-1.717)
Quartile 3	132 (28.9%)	2.267 (1.634-3.145)^*^	1.877 (1.340-2.629)^*^	1.401 (0.938-2.094)	40 (21.7%)	1.191 (0.739-1.921)	1.368 (0.842-2.224)	1.090 (0.652-1.820)
Quartile 4	175 (38.4%)	3.517 (2.555-4.843)^*^	2.901 (2.083-4.040)^*^	**1.778 (1.192-2.653)^#^**	75 (40.8%)	2.459 (1.600-3.778)^*^	2.929 (1.876-4.573)^*^	**2.091 (1.302-3.357)^#^**
*P* for trend	**<0.001**	**<0.001**	**<0.001**	**0.002**	**<0.001**	**<0.001**	**<0.001**	**<0.001**

Logistic regression analyses were used to test the risk of albuminuria, model 1 adjusted for age and sex; model 2 is model 1 further plus exercise, smoking, alcohol consumption. SBP, serum creatinine. *P < 0.001; #*P*<0.05. SBP, systolic blood pressure; LAP, lipid accumulation product; VAI, visceral adiposity index; TyG index, triglyceride-glucose index.

Statistically values are presented in bold.

**Figure 2:**
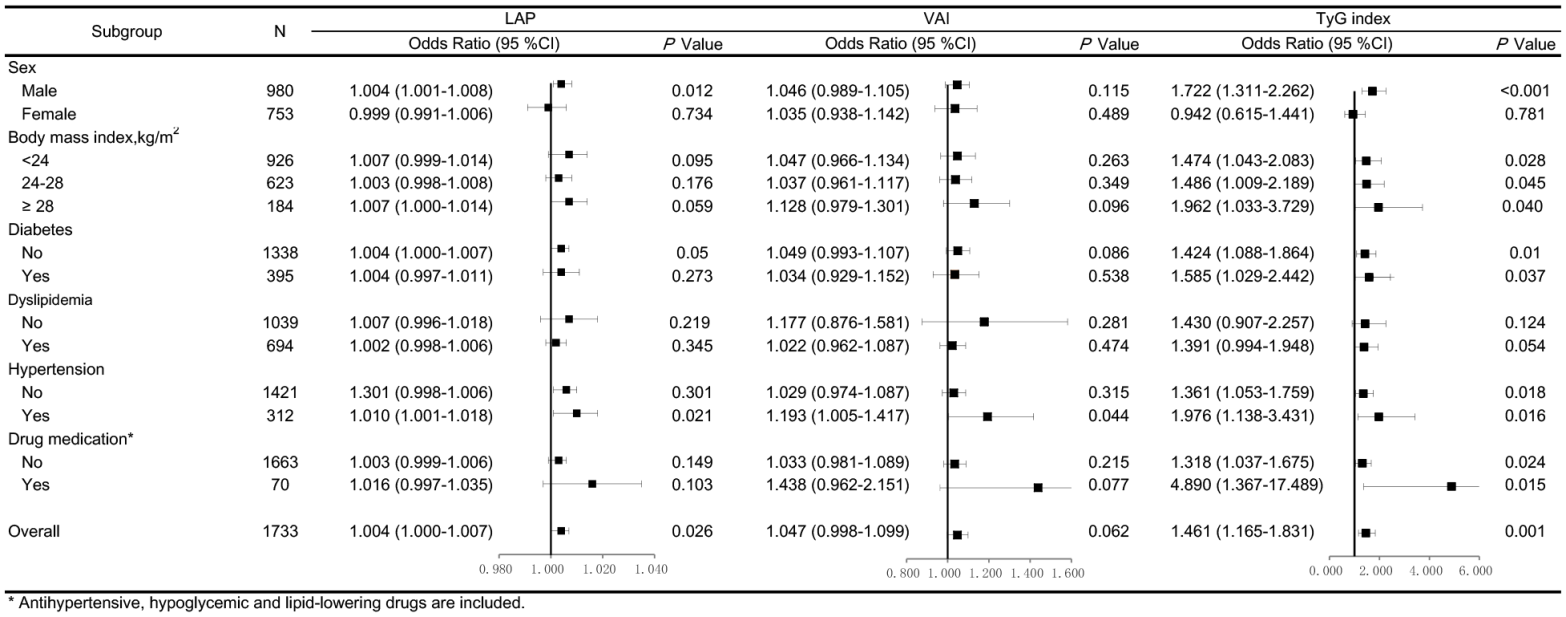
Forest plots of odds ratios (ORs) for the lipid accumulation product (LAP), visceral adiposity index (VAI), and triglyceride-glucose (TyG) index and risk of arterial stiffness after adjustment. The adjustment model includes age, sex, SBP, serum creatinine, physical activity, smoking and alcohol consumption in participants stratified by sex, body mass index, diabetes, dyslipidemia, hypertension, and drug medication. Values are the ORs (95% confidence intervals [95% CIs]).

In addition, the adjusted ORs for arterial stiffness associated with LAP, VAI, and TyG index according to the various subgroups are shown in **Figure 2**. LAP, VAI, and TyG index had better predictive values for arterial stiffness risk in men than in women. Moreover, the relationship between TyG index and the risk of arterial stiffness was consistently observed across all BMI groups, regardless of the presence of cardiometabolic risk factors (diabetes and hypertension) and drug medication (antihypertensive, hypoglycemic, and lipid-lowering drugs).

LAP, VAI, and TyG index had considerable discriminative and calibrating abilities for predicting arterial stiffness, with individual C-index values of 0.856, 0.856, and 0.857, respectively (all *P* < 0.001). According to the IDI analysis, replacing LAP or VAI with the TyG index improved the risk prediction in the fully adjusted model (TyG index *vs.* LAP: IDI [95% CI] = 0.003 [−0.0001 to 0.005], *P* = 0.038; VAI *vs.* TyG index: IDI [95% CI] = −0.003 [−0.006 to 0.0005], *P*=0.019). However, continuous NRI analysis did not show any significant improvement in the prediction (**Table 4**).

**Table 4:** The incremental predictive value of LAP, VAI, and TyG index the for subclinical organ damage in 2017 (n=1,733).

	C-index (95%CI)	*P*	Continuous NRI (95% CI)	*P*	IDI (95% CI)	*P*
Arterial stiffness						
LAP	0.856 (0.837-0.875)	0.983^*^	0.042 (0.016-0.067) ^*^	0.051 ^*^	0.006 (-0.0007 to 0.002) ^*^	0.346 ^*^
VAI	0.856 (0.836-0.875)	0.916*^#^*	-0.037 (-0.094 to 0.024) ***^#^***	0.087 ***^#^***	-0.003 (-0.006 to 0.0005) ***^#^***	**0.019*^#^***
TyG index	0.857 (0.838-0.876)	0.941*^&^*	0.018 (-0.035 to 0.070) ***^&^***	0.486 ***^&^***	0.003 (-0.0001 to 0.005) ***^&^***	**0.038*^&^***
Albuminuria						
LAP	0.739 (0.698-0.781)	0.946^*^	0.103 (0.037-0.176) ^*^	0.021^*^	0.008 (-0.000 to -0.014) ^*^	0.001^*^
VAI	0.737 (0.696-0.778)	0.759*^#^*	-0.109 (-0.211 to 0.000) ***^#^***	**0.039*^#^***	-0.015 (-0.022 to -0.007) ***^#^***	**<0.001*^#^***
TyG index	0.746 (0.706-0.787)	0.811*^&^*	0.005 (-0.091 to 0.097) ***^&^***	0.902 ***^&^***	0.006 (0.0002-0.013) ***^&^***	**0.043*^&^***

Models were adjusted for age, sex, smoking, alcohol consumption, physical activity, systolic blood pressure, and serum creatinine. ^*^ *vs*. VAI, **^#^** *vs*. TyG index, **^&^** *vs*. LAP. 95% CI, 95% confidence intervals; C-index, concordance index; NRI, net reclassification improvement; IDI, integrated discrimination improvement; LAP, lipid accumulation product; VAI, visceral adiposity index; TyG index, triglyceride-glucose index.

Statistically values are presented in bold.

### 3.2 Association of LAP, VAI, and TyG index with albuminuria risk

A total of 184 individuals had albuminuria, and with a uACR value of 63.05 (38.77–138.60) mg/g (**Table S1**). Furthermore, participants with albuminuria had higher LAP, VAI, TyG index, heart rate, SBP, DBP, FBG, TG, baPWV, and Cornell product values and higher proportion of hypertension than those without albuminuria (*P <*0.05).

**Figure 3:**
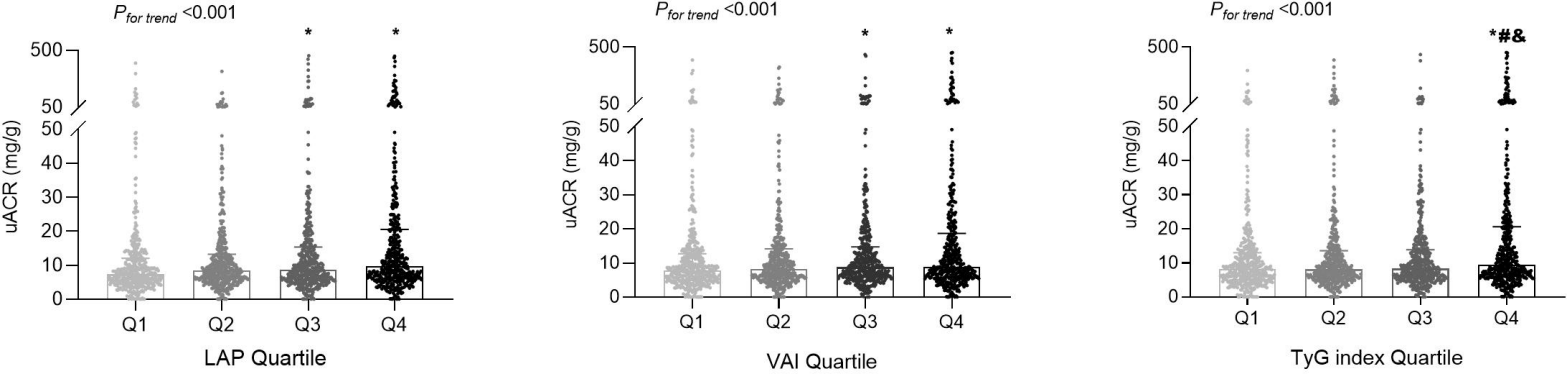
The distributions of the urinary albumin-to-creatinine ratio (uACR) values among the quartile (Q) groups according to the lipid accumulation product (LAP), visceral adiposity index (VAI), and triglyceride-glucose (TyG) index. ^*^P < 0.05 compared with the Q1 group; *^#^P* < 0.05 compared with the Q2 group; ^&^ *P* < 0.05 compared with the Q3 group.

LAP, VAI, and TyG index demonstrated significant positive correlations with uACR value (all *P* < 0.001). As shown in **Figure 3**, the fourth quartiles of LAP, VAI, and TyG index exhibited significantly higher uACRs than the first quartiles (*P* < 0.05). Additionally, LAP (OR [95% CI] = 1.006 [1.003–1.010], *P* < 0.001), VAI (OR [95% CI] = 1.068 [1.015–1.124], *P* = 0.012), and TyG index (OR [95% CI] = 1.861 [1.427–2.426], *P* < 0.001) were independently associated with a higher risk for albuminuria, after adjusting for age, sex, SBP, serum creatinine, physical activity, smoking habit, and alcohol consumption (**Table 2**). Moreover, the high predictive values of the fourth quartiles of LAP and TyG index for the presence of albuminuria were maintained when compared with the first quartiles of LAP and TyG index (OR [95% CI] =1.889 [1.143–3.122], *P* = 0.013; OR [95% CI] = 2.091 [1.302–3.357], *P* = 0.002), respectively, after full adjustment (**Table 3** **)**.

Further examination of albuminuria risk by the three indices according to the various subgroups demonstrated results similar to those for arterial stiffness (**Figure S2**). In all subgroups, the relationship between TyG index and albuminuria risk was significantly stronger than that between LAP and VAI and the risk of albuminuria (*P* < 0.05 for both).

As displayed in **Table 4**, further predictive performance evaluation revealed that LAP, VAI, and TyG index had C-index values of 0.739, 0.737, and 0.746 (all *P* < 0.001), respectively, indicating good discrimination by all three indices. Based on the IDI analysis, the TyG index significantly improved the discrimination of albuminuria risk (TyG index *vs.* LAP: IDI [95% CI] = 0.006 [0.0002–0.013], *P* = 0.043 and VAI *vs.* TyG index: IDI [95% CI] = −0.015 [−0.022 to −0.007], *P* < 0.001). Furthermore, continuous NRI analysis revealed that the TyG index significantly improved the discrimination of albuminuria risk compared with VAI.

### 3.3 Association of LAP, VAI, and TyG index with LVH risk

The characteristics of participants with and without ECG-LVH are presented in **Table S2**, with a median Cornell product value of 136.56 mV·ms (without ECG-LVG) and 280.24 mV·ms (with ECG-LVG). Participants with ECG-LVH (n = 84) had a higher prevalence of hypertension and higher BMI, WC, SBP, DBP, and uACR values than those without ECG-LVH (*P* < 0.05). However, LAP, VAI, and TyG index values were not significantly different between the two groups. In addition, LAP and VAI values were positively correlated with Cornell product (*P* < 0.05 for both), but these associations were not detected when the LAP and VAI index values were divided into quartiles (**Figure S3**).

## 4 Discussion

In this study, we found that elevated values of LAP, VAI, and TyG index were independently associated with a higher risk of arterial stiffness and albuminuria but not with the risk of ECG-LVH. Additionally, the TyG index exhibited superior performance in identifying arterial stiffness and albuminuria compared with the other two indices. Thus, our findings suggest that these three markers of visceral adiposity, especially the TyG index, may serve as simple and noninvasive indicators to predict cardiovascular SOD in clinical practice.

Prior literature has suggested that the novel parameters of visceral adiposity, namely, LAP, VAI, and TyG index, are associated with baPWV. In a study involving Chinese hypertensive patients with a mean age of 64.42 years, a positive association was found between LAP and elevated baPWV (>75^th^ percentile) (36). Another study reported that older Chinese participants (>60 years) in the higher VAI tertiles had a higher odds ratio (OR) for arterial stiffness (defined as baPWV ≥1,400 cm/s) compared with those in the lowest VAI tertile (37). In the Kailuan study, a significant dose–response relationship between the TyG index and the risk of arterial stiffness, measured by baPWV, was observed (38). However, the comparative predictive capabilities of LAP, VAI, and TyG index for arterial stiffness in the general population remain unclear. Our study revealed that all high quartiles of LAP, VAI, and TyG index were significantly associated with an increased risk of arterial stiffness after adjusting for confounding factors. Furthermore, our findings demonstrated that the TyG index outperformed the other two indices in all subgroup analyses. Therefore, our results indicate that the three parameters, especially the TyG index, possess considerable potential as simple and effective markers for identifying individuals with a high risk of vascular dysfunction.

Our study is the first to evaluate and compare the associations between all three parameters, namely, LAP, VAI, and TyG index, and albuminuria in the general Chinese population. Although previous studies have shown strong associations between obesity-related indices and chronic kidney disease (39), the findings are inconsistent. A cohort study involving 1,872 patients with type 2 diabetes reported a higher risk of albuminuria associated with elevated LAP, VAI, and TyG index (40). However, a community cohort study of 3,868 participants followed up for over 3.1 years revealed that albuminuria incidence increased proportionally with TyG index quartiles, but the TyG index itself was not identified as an independent risk factor for albuminuria (41). In our study, LAP, VAI, and TyG index were independently associated with an increased risk of albuminuria. Furthermore, our findings indicate that the TyG index outperformed the other two indices in all subgroup analyses, regardless of the presence of cardiometabolic risk factors and drug medication.

Albuminuria is a systemic vascular disturbance that is potentially associated with increased cardiovascular morbidity and mortality in obese individuals (42). The distribution of fat in the body may influence the health of arteries and kidneys (43). In particular, visceral adipose tissue can regulate pro-inflammatory cytokines, such as interleukin 6, and reduce the production of adiponectin, a cardiovascular protective protein. This contributes to inflammation, oxidative stress, insulin resistance, and podocyte dysfunction. These alterations can result in arterial stiffness, albuminuria, and other cardiovascular risks (44, 45). Additionally, excessive fat infiltration in the kidneys can further worsen renal damage (46).

Visceral adiposity can also influence cardiac remodeling via underlying mechanisms described above. A cohort study of 229 participants with suspected metabolic syndrome aged 56.4 ± 4.5 years demonstrated that visceral obesity, but not central obesity measured by WC, was independently associated with structural and functional cardiac remodeling (47). However, our current study of the general population, with a mean age of 43 (40–45) years, did not find any association between adiposity indices (LAP, VAI, and TyG index) and ECG-LVH. It would be necessary to observe long-term cardiac structural changes, since a large number of older individuals in the outcome group of the general population could help clarify the association between obesity indices and subclinical heart damage. However, we did confirm the findings of a previous investigation reporting an association between obesity phenotypes and LVH (48). Obesity is increasingly recognized as a heterogeneous condition with a cluster of metabolic derangements postulated to explain its association with cardiovascular organ dysfunction (27, 46). Therefore, we hypothesize that visceral adipose tissue and ectopic fat depots may play a significant role in the prevalence and progression of SOD.

The main advantages of this study were that it explored and compared the associations between three obesity-related indices and SOD in a general Chinese population. Additionally, the study aimed to investigate a wide range of subclinical organ outcomes, including blood vessels, kidneys, and the heart, instead of focusing on just one aspect. This comprehensive approach allows for a holistic evaluation of the cardiovascular high-risk group within this population. Furthermore, SOD outcomes were collected by a panel of physicians using detailed evaluation criteria, and the standardized data collection protocols and rigorous quality control. Nonetheless, some limitations merit consideration. First, our study was a cross-sectional analysis; therefore, a causal relationship between the three obesity indices and SOD outcomes could not be established. Second, the formula to calculate LAP was obtained by Kahn based on the data of the National Nutrition Survey of the United States (13). The parameter settings are accordingly derived from the Western population. Therefore, the formula’s applicability to the Chinese population requires further exploration. Similar validations are necessary for VAI and TyG index as well. Notably, there is growing evidence of the value of the three markers in East Asians. Third, our study results should be confirmed by epidemiological data from other regions and larger populations.

In conclusion, LAP, VAI, and particularly TyG index can help identify SOD in clinical settings and stratify the high-risk group requiring early prevention strategies. Individuals with a higher TyG index have an elevated risk for vascular failure and early kidney damage in Chinese Han adults. These findings will help implement early detection approaches and preventive measures against cardiovascular SOD progression and adverse cardiovascular outcomes.

## Data availability statement

The data that support the findings of this study are available from the corresponding author upon reasonable request. Requests to access the datasets should be directed to YW, wangyangxxk@126.com.

## Ethics statement

The studies involving humans were approved by the Ethics Committee of the First Affiliated Hospital of Xi’an Jiaotong University (XJTU1AF2015LSL-047). The studies were conducted in accordance with the local legislation and institutional requirements. The participants provided their written informed consent to participate in this study.

## Author contributions

YW and M-FD conceived and designed the experiments; J-JM was responsible for participant recruitment; M-FD, XZ, G-LH, CChu, Y-YL, CChen, DW, QM, YY, HJ, K-KW, YS, Z-JN, Z-YM, LW, X-YZ, W-JL, W-HG, HL, G-JW, KG, and JZ performed the experiments; M-FD and YW analyzed the data; M-FD drafted the manuscript; and YW and J-JM edited and revised the manuscript. All authors contributed to the article and approved the submitted version.

## References

[1] Liu S Li Y Zeng X Wang H Yin P Wang L. Burden of cardiovascular diseases in China, 1990-2016: findings from the 2016 global burden of disease study. JAMA Cardiol (2019) 4:342–52. doi: 10.1001/jamacardio.2019.0295 PMC6484795 30865215

[2] Tsuboi N. Obesity indices and the risk of CKD. Intern Med (2021) 60:1987–8. doi: 10.2169/internalmedicine.6921-20 PMC8313901 33551414

[3] Chang AR Grams ME Ballew SH Bilo H Correa A Evans M. Adiposity and risk of decline in glomerular filtration rate: meta-analysis of individual participant data in a global consortium. BMJ (2019) 364:k5301. doi: 10.1136/bmj.k5301 30630856 PMC6481269

[4] Alpert MA Karthikeyan K Abdullah O Ghadban R. Obesity and cardiac remodeling in adults: mechanisms and clinical implications. Prog Cardiovasc Dis (2018) 61:114–23. doi: 10.1016/j.pcad.2018.07.012 29990533

[5] Sutton-Tyrrell K Najjar SS Boudreau RM Venkitachalam L Kupelian V Simonsick EM. Elevated aortic pulse wave velocity, a marker of arterial stiffness, predicts cardiovascular events in well-functioning older adults. Circulation (2005) 111:3384–90. doi: 10.1161/CIRCULATIONAHA.104.483628 15967850

[6] Fox CS Massaro JM Hoffmann U Pou KM Maurovich-Horvat P Liu CY. Abdominal visceral and subcutaneous adipose tissue compartments: association with metabolic risk factors in the Framingham Heart Study. Circulation (2007) 116:39–48. doi: 10.1161/CIRCULATIONAHA.106.675355 17576866

[7] Wisse BE. The inflammatory syndrome: the role of adipose tissue cytokines in metabolic disorders linked to obesity. J Am Soc Nephrol (2004) 15:2792–800. doi: 10.1097/01.ASN.0000141966.69934.21 15504932

[8] Neeland IJ Ross R Després JP Matsuzawa Y Yamashita S Shai I. Visceral and ectopic fat, atherosclerosis, and cardiometabolic disease: a position statement. Lancet Diabetes Endocrinol (2019) 7:715–25. doi: 10.1016/S2213-8587(19)30084-1 31301983

[9] Oh JY Sung YA Lee HJ. The visceral adiposity index as a predictor of insulin resistance in young women with polycystic ovary syndrome. Obes (Silver Spring) (2013) 21:1690–4. doi: 10.1002/oby.20096 23585246

[10] Hiuge-Shimizu A Kishida K Funahashi T Ishizaka Y Oka R Okada M. Absolute value of visceral fat area measured on computed tomography scans and obesity-related cardiovascular risk factors in large-scale Japanese general population (the VACATION-J study). Ann Med (2012) 44:82–92. doi: 10.3109/07853890.2010.526138 20964583

[11] Li XH Feng ST Cao QH Coffey JC Baker ME Huang L. Degree of creeping fat assessed by computed tomography enterography is associated with intestinal fibrotic stricture in patients with crohn's disease: A potentially novel mesenteric creeping fat index. J Crohns Colitis (2021) 15(7):1161–73. doi: 10.1093/ecco-jcc/jjab005 PMC8427713 33411893

[12] Hoenig MR. MRI sagittal abdominal diameter is a stronger predictor of metabolic syndrome than visceral fat area or waist circumference in a high-risk vascular cohort. Vasc Health Risk Manage (2010) 6:629–33. doi: 10.2147/vhrm.s10787 PMC2922324 20730019

[13] Kahn HS. The "lipid accumulation product" performs better than the body mass index for recognizing cardiovascular risk: a population-based comparison. BMC Cardiovasc Disord (2005) 5:26. doi: 10.1186/1471-2261-5-26 16150143 PMC1236917

[14] Kahn HS. The lipid accumulation product is better than BMI for identifying diabetes: a population-based comparison. Diabetes Care (2006) 29:151–3. doi: 10.2337/diacare.29.1.151 16373916

[15] Zhang Y Hu J Li Z Li T Chen M Wu L. A novel indicator of lipid accumulation product associated with metabolic syndrome in chinese children and adolescents. Diabetes Metab Syndr Obes (2019) 12:2075–83. doi: 10.2147/DMSO.S221786 PMC6791402 31632117

[16] Huang J Bao X Xie Y Zhang X Peng X Liu Y. Interaction of lipid accumulation product and family history of hypertension on hypertension risk: a cross-sectional study in the Southern Chinese population. BMJ Open (2019) 9:e029253. doi: 10.1136/bmjopen-2019-029253 PMC6924775 31784431

[17] Hosseinpanah F Barzin M Mirbolouk M Abtahi H Cheraghi L Azizi F. Lipid accumulation product and incident cardiovascular events in a normal weight population: Tehran Lipid and Glucose Study. Eur J Prev Cardiol (2016) 23:187–93. doi: 10.1177/2047487314558771 25381336

[18] Mousapour P Barzin M Valizadeh M Mahdavi M Azizi F Hosseinpanah F. Predictive performance of lipid accumulation product and visceral adiposity index for renal function decline in non-diabetic adults, an 8. 6-year Follow-up Clin Exp Nephrol (2020) 24:225–34. doi: 10.1007/s10157-019-01813-7 31734819

[19] Amato MC Giordano C Galia M Criscimanna A Vitabile S Midiri M. Visceral Adiposity Index: a reliable indicator of visceral fat function associated with cardiometabolic risk. Diabetes Care (2010) 33:920–2. doi: 10.2337/dc09-1825 PMC2845052 20067971

[20] Yan Y Wang D Sun Y Ma Q Wang K Liao Y. Triglyceride-glucose index trajectory and arterial stiffness: results from Hanzhong Adolescent Hypertension Cohort Study. Cardiovasc Diabetol (2022) 21:33. doi: 10.1186/s12933-022-01453-4 35216614 PMC8876112

[21] Barzegar N Tohidi M Hasheminia M Azizi F Hadaegh F. The impact of triglyceride-glucose index on incident cardiovascular events during 16 years of follow-up: Tehran Lipid and Glucose Study. Cardiovasc Diabetol (2020) 19:155. doi: 10.1186/s12933-020-01121-5 32993633 PMC7526412

[22] Yan Y Zheng W Ma Q Chu C Hu J Wang K. Child-to-adult body mass index trajectories and the risk of subclinical renal damage in middle age. Int J Obes (Lond) (2021) 45:1095–104. doi: 10.1038/s41366-021-00779-5 33608649

[23] Liao YY Ma Q Chu C Wang Y Zheng WL Hu JW. The predictive value of repeated blood pressure measurements in childhood for cardiovascular risk in adults: the Hanzhong Adolescent Hypertension Study. Hypertens Res (2020) 43:969–78. doi: 10.1038/s41440-020-0480-7 32488216

[24] Wang Y Yuan Y Gao WH Yan Y Wang KK Qu PF. Predictors for progressions of brachial-ankle pulse wave velocity and carotid intima-media thickness over a 12-year follow-up: Hanzhong Adolescent Hypertension Study. J Hypertens (2019) 37:1167–75. doi: 10.1097/HJH.0000000000002020 PMC6513272 31026243

[25] Ma YC Zuo L Chen JH Luo Q Yu XQ Li Y. Modified glomerular filtration rate estimating equation for Chinese patients with chronic kidney disease. J Am Soc Nephrol. (2006) 17:2937–44. doi: 10.1681/ASN.2006040368 16988059

[26] Ahn N Baumeister SE Amann U Rathmann W Peters A Huth C. Visceral adiposity index (VAI), lipid accumulation product (LAP), and product of triglycerides and glucose (TyG) to discriminate prediabetes and diabetes. Sci Rep (2019) 9:9693. doi: 10.1038/s41598-019-46187-8 31273286 PMC6609728

[27] Nusrianto R Ayundini G Kristanti M Astrella C Amalina N Muhadi. Visceral adiposity index and lipid accumulation product as a predictor of type 2 diabetes mellitus: The Bogor cohort study of non-communicable diseases risk factors. Diabetes Res Clin Pract (2019) 155:107798. doi: 10.1038/s41598-019-46187-8 31330161

[28] Mancia Chairperson G Kreutz Co-Chair R Brunström M Burnier M Grassi G Januszewicz A. ESH Guidelines for the management of arterial hypertension The Task Force for the management of arterial hypertension of the European Society of Hypertension Endorsed by the European Renal Association (ERA) and the International Society of Hypertension (ISH) [published online ahead of print, 2023 Jun 21]. J Hypertens (2023). doi: 10.1097/HJH.0000000000003480 37345492

[29] Jia W Weng J Zhu D Ji L Lu J Zhou Z. Standards of medical care for type 2 diabetes in China 2019. Diabetes Metab Res Rev (2019) 35:e3158. doi: 10.1002/dmrr.3158 30908791

[30] Joint Committee on the Chinese Guidelines for Lipid Management. Chinese guidelines for lipid management (2023). Zhonghua Xin Xue Guan Bing Za Zhi (2023) 51(3):221–55. doi: 10.3760/cma.j.cn112148-20230119-00038 36925135

[31] Chinese Society of Endocrinology, Chinese Medical Association. Guideline for the diagnosis and management of hyperuricemia and gout in China (2019). Chin J Endocrinol Metab (2020) 36(01):1–13. doi: 10.3760/cma.j.issn.1000-6699.2020.01.001

[32] Ikizler TA Burrowes JD Byham-Gray LD Campbell KL Carrero JJ Chan W. KDOQI clinical practice guideline for nutrition in CKD: 2020 update. Am J Kidney Dis (2020) 76:S1–1S107. doi: 10.1053/j.ajkd.2020.05.006 32829751

[33] Tanaka A Tomiyama H Maruhashi T Matsuzawa Y Miyoshi T Kabutoya T. Physiological diagnostic criteria for vascular failure. Hypertension (2018) 72:1060–71. doi: 10.1161/HYPERTENSIONAHA.118.11554 30354826

[34] Schillaci G Battista F Pucci G. A review of the role of electrocardiography in the diagnosis of left ventricular hypertrophy in hypertension. J Electrocardiol (2012) 45:617–23. doi: 10.1016/j.jelectrocard.2012.08.051 23022303

[35] Hosmer DW Lemeshow S. Applied Logistic Regression (2nd Edition). New York: John Wiley & Sons (2000). doi: 10.1080/00401706.1992.10485291

[36] Shi Y Hu L Li M Zhou W Wang T Zhu L. Relationship between the lipid accumulation product index and arterial stiffness in the chinese population with hypertension: A report from the China H-type hypertension registry study. Front Cardiovasc Med (2021) 8:760361. doi: 10.3389/fcvm.2021.760361 35146005 PMC8823664

[37] Fan Y Wang Z Zhao X Wu S Chi H. Association of the visceral adiposity index with arterial stiffness in elderly Chinese population. Am J Med Sci (2023) 365(3):279–285. doi: 10.1016/j.amjms.2022.10.010 36335991

[38] Wu S Xu L Wu M Chen S Wang Y Tian Y. Association between triglyceride-glucose index and risk of arterial stiffness: a cohort study. Cardiovasc Diabetol (2021) 20:146. doi: 10.1186/s12933-021-01342-2 34271940 PMC8285795

[39] Chen T Wang X Wang X Chen H Xiao H Tang H. Comparison of novel metabolic indices in estimation of chronic kidney diseases in a southern Chinese population. Diabetes Metab Syndr Obes (2020) 13:4919–27. doi: 10.2147/DMSO.S286565 PMC7735784 33328750

[40] Ou YL Lee MY Lin IT Wen WL Hsu WH Chen SC. Obesity-related indices are associated with albuminuria and advanced kidney disease in type 2 diabetes mellitus. Ren Fail (2021) 43:1250–8. doi: 10.1080/0886022X.2021.1969247 PMC8409948 34461808

[41] Xu X Tang X Che H Guan C Zhao N Fu S. Triglyceride-glucose product is an independent risk factor for predicting chronic kidney disease in middle-aged and elderly population: a prospective cohort study. Nan Fang Yi Ke Da Xue Xue Bao (2021) 41:1600–8. doi: 10.12122/j.issn.1673-4254.2021.11.02 PMC8685706 34916184

[42] Bahrami H Bluemke DA Kronmal R Bertoni AG Lloyd-Jones DM Shahar E. Novel metabolic risk factors for incident heart failure and their relationship with obesity: the MESA (Multi-Ethnic Study of Atherosclerosis) study. J Am Coll Cardiol (2008) 51:1775–83. doi: 10.1016/j.jacc.2007.12.048 18452784

[43] Arner P Bäckdahl J Hemmingsson P Stenvinkel P Eriksson-Hogling D Näslund E. Regional variations in the relationship between arterial stiffness and adipocyte volume or number in obese subjects. Int J Obes (Lond) (2015) 39:222–7. doi: 10.1038/ijo.2014.118 25002147

[44] Tao L Gao E Jiao X Yuan Y Li S Christopher TA. Adiponectin cardioprotection after myocardial ischemia/reperfusion involves the reduction of oxidative/nitrative stress. Circulation (2007) 115:1408–16. doi: 10.1161/CIRCULATIONAHA.106.666941 17339545

[45] Sharma K Ramachandrarao S Qiu G Usui HK Zhu Y Dunn SR. Adiponectin regulates albuminuria and podocyte function in mice. J Clin Invest (2008) 118:1645–56. doi: 10.1172/JCI32691 PMC2323186 18431508

[46] Wahba IM Mak RH. Obesity and obesity-initiated metabolic syndrome: mechanistic links to chronic kidney disease. Clin J Am Soc Nephrol (2007) 2:550–62. doi: 10.2215/CJN.04071206 17699463

[47] Cho DH Kim MN Joo HJ Shim WJ Lim DS Park SM. Visceral obesity, but not central obesity, is associated with cardiac remodeling in subjects with suspected metabolic syndrome. Nutr Metab Cardiovasc Dis (2019) 29:360–6. doi: 10.1016/j.numecd.2019.01.007 30782509

[48] Ahmad MI Li Y Soliman EZ. Association of obesity phenotypes with electrocardiographic left ventricular hypertrophy in the general population. J Electrocardiol (2018) 51:1125–30. doi: 10.1016/j.jelectrocard.2018.10.085 30497743

