# Supplementary material for: Associations of lipid accumulation product, visceral adiposity index, and triglyceride-glucose index with subclinical organ damage in healthy Chinese adults
Source: Front Endocrinol (Lausanne). 2023 Sep 19;14:1164592. doi: 10.3389/fendo.2023.1164592 (PMC10546403; doi:10.3389/fendo.2023.1164592)
Supplement: Supplementary file 1 [file DataSheet_1.docx]

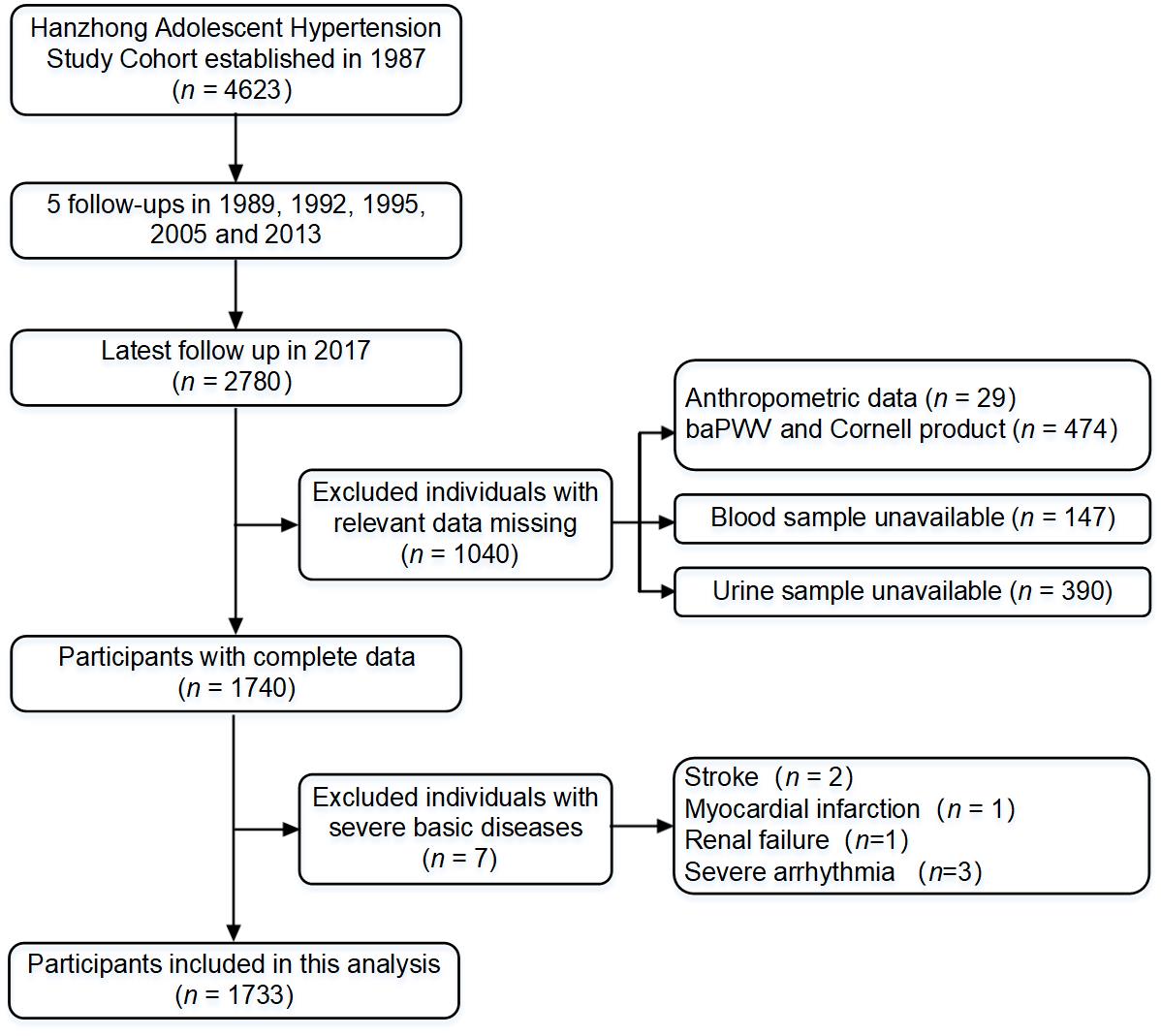


**Fig. S1** Flowchart for inclusion/exclusion of study participants

**
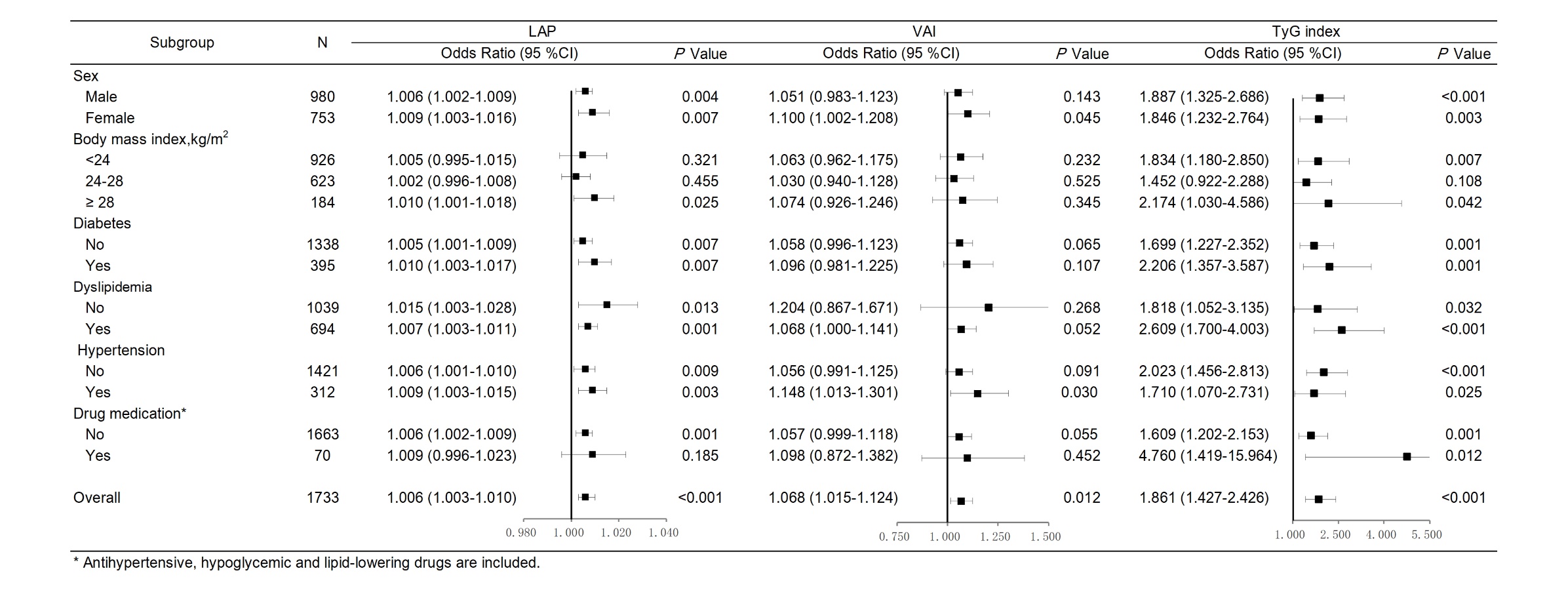
**

**Fig. S2** Forest plots of odds ratios (95% confidence intervals) [ORs (95% CIs)] for lipid accumulation product (LAP), visceral adiposity index (VAI) and triglyceride-glucose (TyG) index and risk of albuminuria after adjustment. The adjustment model includes age, sex, SBP, serum creatinine, physical activity, smoking and alcohol consumption in subjects stratified by sex, body mass index, diabetes mellitus, dyslipidemia, hypertension and drug medication

**
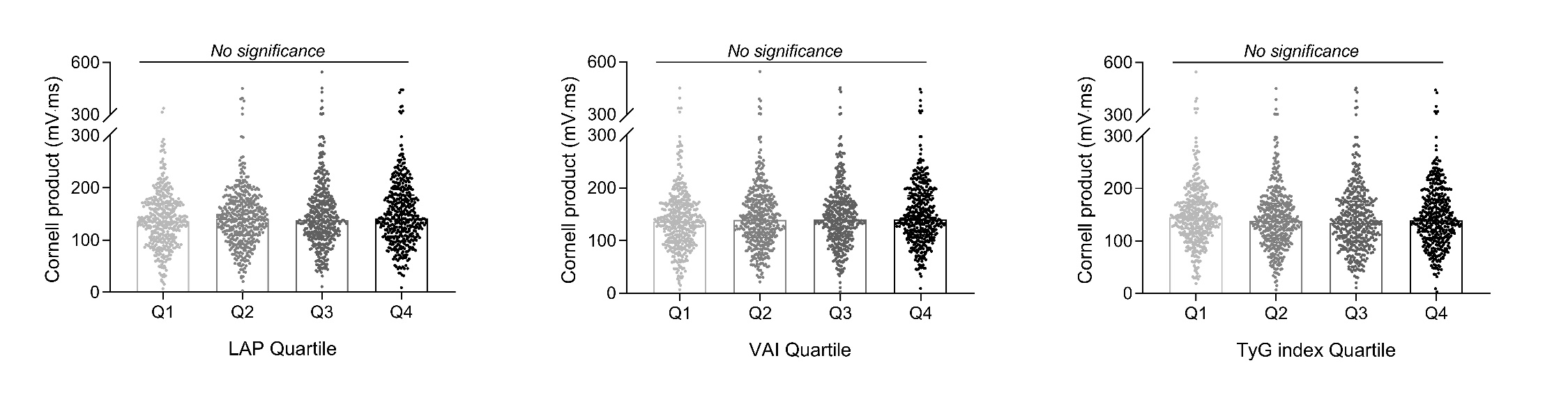
**

**Fig. S3** The distributions of the Cornell product value among the quartile (Q) groups according to the lipid accumulation product (LAP), visceral adiposity index (VAI) and triglyceride-glucose (TyG) index

**Table S1.** Characteristics of participants categorized by albuminuria status in 2017 (n = 1733).

| Characteristics | All (n = 1733) | Non-albuminuria (n = 1549) | Albuminuria (n = 184) | *P* value |
| --- | --- | --- | --- | --- |
| Gender (Male, %) | 980 (56.55%) | 889 (57.39%) | 91 (49.46%) | **0.040** |
| Age (years) | 43 (40-45) | 43 (40-45) | 42 (40-45) | 0.192 |
| Diabetes mellitus (%) | 395 (22.79%) | 345 (22.25%) | 50 (27.17%) | 0.134 |
| Hypertension (%) | 312 (18.00%) | 244 (15.75%) | 68 (36.96%) | **<0.001** |
| Hyperuricemia (%) | 89 (5.14%) | 76 (4.91%) | 13 (7.07%) | 0.210 |
| Hyperlipidemia (%) | 694 (40.05%) | 612 (39.51%) | 82 (44.57%) | 0.186 |
| Alcohol consumption (%) | 509 (29.37%) | 467 (30.15%) | 42 (22.83%) | **0.039** |
| Current smoking (%) | 757 (43.68%) | 679 (43.83%) | 78 (42.39%) | 0.709 |
| Obesity indices | | | | |
| BMI (kg/m^2^) | 23.78 (21.85-26.02) | 23.64 (21.75-25.80) | 25.29 (22.92-27.57) | **<0.001** |
| WC (cm) | 84.20 (78.00-91.40) | 83.80 (77.90-90.90) | 88.58 ± 11.15 | **<0.001** |
| LAP | 29.77 (17.22-50.69) | 28.80 (16.81-48.22) | 39.60 (22.21-76.35) | **<0.001** |
| VAI | 1.80 (1.17-2.76) | 1.77 (1.15-2.68) | 2.05 (1.39-3.90) | **<0.001** |
| TyG index | 8.49 (8.14-8.90) | 8.48 (8.13-8.87) | 8.81 ± 0.75 | **<0.001** |
| Measurement indicators | | | | |
| Heart rate (beats/min) | 73 (66-80) | 73 (66-79) | 77 (70-84) | **<0.001** |
| SBP (mmHg) | 121 (112 -131) | 120 (112-130) | 131 (120-149) | **<0.001** |
| DBP (mmHg) | 76 (69-84) | 75 (69-83) | 85 ± 15 | **<0.001** |
| FBG (mmol/L) | 4.57 (4.28-4.90) | 4.56 (4.28-4.87) | 4.72 (4.35-5.19) | **<0.001** |
| ALT(U/L) | 19 (14-27) | 19 (14-27) | 19 (14-30) | 0.344 |
| AST(U/L) | 16 (13-20) | 16 (13-20) | 16 (13-22) | 0.928 |
| Total cholesterol (mmol/L) | 4.50 (4.04-5.01) | 4.49 (4.03-5.00) | 4.64 (4.12-5.15) | 0.054 |
| Triglycerides (mmol/L) | 1.34 (0.95-1.94) | 1.33 (0.94-1.90) | 1.57 (1.03-2.45) | **<0.001** |
| LDL-C (mmol/L) | 2.50 (2.12-2.91) | 2.50 (2.12-2.89) | 2.50 (2.10-3.04) | 0.610 |
| HDL-C (mmol/L) | 1.15 (0.99-1.33) | 1.15 (0.99-1.33) | 1.18 ± 0.26 | 0.135 |
| SUA (μmol/L) | 279.90 (225.00-336.10) | 279.50 (225.20-334.35) | 284.25 (221.75-334.13) | 0.555 |
| Serum creatinine (μmol/L) | 75.60 (66.45-85.90) | 75.80 (66.50-86.05) | 74.70 (64.93-85.13) | 0.277 |
| eGFR (mL/min/1.73m^2^) | 97.76 (87.60-110.77) | 97.78 (87.89-110.48) | 97.56 (84.26-116.69) | 0.865 |
| uACR (mg/g) | 8.45 (5.52-14.72) | 7.70 (5.23-12.06) | 63.05 (38.77-138.60) | **<0.001** |
| baPWV (cm/s) | 1263.00 (1126.00-1415.50) | 1248.00 (1119.00-1391.50) | 1422.50 (1232.25-1607.25) | **<0.001** |
| Cornell product (mV·ms) | 138.60 (106.62-174.88) | 136.71 (105.30-172.80) | 158.25 (125.56-199.58) | **<0.001** |

Non-normally distributed variables are expressed as the median (interquartile range). All other values are expressed as mean ± SD or n (%).BMI, body mass index; WC, waist circumference; LAP, lipid accumulation product; VAI, visceral adiposity index; TyG index, triglyceride-glucose index; SBP, systolic blood pressure; DBP, diastolic blood pressure; FBG, fasting blood glucose; ALT, alanine aminotransferase; AST, aspartate aminotransferase; LDL-C, low-density lipoprotein cholesterol; HDL-C, high-density lipoprotein cholesterol; UA, urine acid; eGFR, estimated glomerular filtration rate; uACR, urine albumin-to creatinine ratio. Statistically values are presented in bold.

**Table S2.** Characteristics of participants categorized by ECG-LVH status in 2017 (n = 1733).

| Characteristics | All (n = 1733) | Non-ECG-LVH (n = 1649) | ECG-LVH (n = 84) | *P* value | | |
| --- | --- | --- | --- | --- | --- | --- |
| Gender (Male, %) | 980 (56.55%) | 941 (57.06%) | 39 (46.43%) | 0.055 | | |
| Age (years) | 43 (40-45) | 43 (40-45) | 43 (41-45) | 0.809 | | |
| Diabetes mellitus (%) | 395 (22.79%) | 374 (22.68%) | 21 (25.00%) | 0.621 | | |
| Hypertension (%) | 312 (18.00%) | 286 (17.34%) | 26 (30.95%) | **0.002** | | |
| Hyperuricemia (%) | 89 (5.14%) | 82 (4.98%) | 7 (8.33%) | 0.173 | | |
| Hyperlipidemia (%) | 694 (40.05%) | 662 (40.15%) | 32 (38.10%) | 0.708 | | |
| Alcohol consumption (%) | 509 (29.37%) | 486 (29.47%) | 23 (27.38%) | 0.681 | | |
| Current smoking (%) | 757 (43.68%) | 724 (43.91%) | 33 (39.29%) | 0.405 | | |
| Obesity indices | | | | | | |
| BMI (kg/m^2^) | 23.78 (21.85-26.02) | 23.72 (21.81-25.95) | 25.00 ± 3.23 | **0.002** | | |
| WC (cm) | 84.20 (78.00-91.40) | 84.00 (78.00-91.15) | 88.58 ± 11.15 | **0.009** | | |
| LAP | 29.77 (17.22-50.69) | 29.52 (17.01-50.39) | 34.76 (20.83-55.73) | 0.098 | | |
| VAI | 1.80 (1.17-2.76) | 1.80 (1.17-2.77) | 1.75 (1.20-2.71) | 0.859 | | |
| TyG index | 8.49 (8.14-8.90) | 8.50 (8.15-8.91) | 8.41 (8.11-8.83) | 0.183 | | |
| Measurement indicators | | | | |  |  |
| Heart rate (beats/min) | 73 (66-80) | 73 (66-80) | 74.96 ± 10.72 | 0.239 | |  |
| SBP (mmHg) | 121 (112 -131) | 121 (112-130) | 129 ± 18 | **0.003** | |  |
| DBP (mmHg) | 76 (69-84) | 76 (69-83) | 82 ± 12 | **<0.001** | |  |
| FBG (mmol/L) | 4.57 (4.28-4.90) | 4.57 (4.28-4.90) | 4.55 (4.26-4.90) | 0.749 | |  |
| ALT(U/L) | 19 (14-27) | 19 (14-27) | 20 (15-30) | 0.086 | |  |
| AST(U/L) | 16 (13-20) | 16 (13-20) | 17 (13-22) | 0.421 | |  |
| Total cholesterol (mmol/L) | 4.50 (4.04-5.01) | 4.49 (4.04-5.02) | 4.55 ± 0.73 | 0.681 | |  |
| Triglycerides (mmol/L) | 1.34 (0.95-1.94) | 1.35 (0.95-1.95) | 1.31 (0.99-1.83) | 0.977 | |  |
| LDL-C (mmol/L) | 2.50 (2.12-2.91) | 2.50 (2.12-2.91) | 2.52 ± 0.63 | 0.936 | |  |
| HDL-C (mmol/L) | 1.15 (0.99-1.33) | 1.14 (0.99-1.33) | 1.20 ± 0.24 | 0.207 | |  |
| SUA (μmol/L) | 279.90 (225.00-336.10) | 280.20 (225.00-335.60) | 278.10 (228.85-346.83) | 0.821 | |  |
| Serum creatintine (μmol/L) | 75.60 (66.45-85.90) | 75.70 (66.65-86.00) | 73.17 ± 15.36 | **0.026** | |  |
| eGFR (mL/min/1.73m^2^) | 97.76 (87.60-110.77) | 97.59 (87.55-110.63) | 104.28 ± 21.13 | **0.036** | |  |
| uACR (mg/g) | 8.45 (5.52-14.72) | 8.33 (5.45-14.50) | 11.09 (6.70-24.66) | **<0.001** | |  |
| baPWV (cm/s) | 1263.00 (1126.00-1415.50) | 1261.00 (1125.00-1415.50) | 1305.55 ± 207.14 | 0.250 | |  |
| Cornell product (mV·ms) | 138.60 (106.62-174.88) | 136.56 ± 46.49 | 280.24 (257.74-312.20) | **<0.001** | |  |

Non-normally distributed variables are expressed as the median (interquartile range). All other values are expressed as mean ± SD or n (%). LVH, left ventricular hypertrophy; BMI, body mass index; WC, waist circumference; LAP, lipid accumulation product; VAI, visceral adiposity index; TyG index, triglyceride-glucose index; SBP, systolic blood pressure; DBP, diastolic blood pressure; FBG, fasting blood glucose; ALT, alanine aminotransferase; AST, aspartate aminotransferase; LDL-C, low-density lipoprotein cholesterol; HDL-C, high-density lipoprotein cholesterol; UA, urine acid; eGFR, estimated glomerular filtration rate; uACR, urine albumin-to creatinine ratio. Statistically values are presented in bold.
